# Une ulcération atypique de la lèvre inférieure

**DOI:** 10.11604/pamj.2017.28.180.13953

**Published:** 2017-10-27

**Authors:** Ilhame Naciri, Karima Senouci

**Affiliations:** 1 Service de Dermatologie et Vénérologie, Centre Hospitalier Universitaire Ibn Sina, Faculté de Médecine et de Pharmacie, Université Mohammed V, Rabat, Maroc

**Keywords:** Pathomimie, lèvre, ulcération, Pathomimia, lip, ulceration

## Abstract

Cutaneous pathomimia is a self-induced disease characterized by conscious self-injury to mucocutaneous boundary or its phaneres. We report the case of a 22-year old woman, with no notable medical history, presenting with sudden onset ulceration on her lower lip evolving for 3 months in a context of preservation of patient’s clinical status. The patient had previously consulted several dermatologists and had undergone two skin biopsies with non-specific histological diagnosis. Clinical examination showed painful, well-defined ulceration with clean surface and non-indurated basis, measuring 1 × 3 cm in diameter and involving the left half of the lower lip (A). Given the clinical appearance of the lesion, its chronic nature and the lack of response to local care, several diagnosis were suspected: squamous cell carcinoma, cutaneous leishmaniasis, pyoderma gangrenosum… Deep biopsy was performed showing a loss of substance revealing the chorion, without histological characters of malignancy. Syphilis and HIV tests were negative. Fifteen days after, patient’s evolution was marked by the appearance of an identical lesion on the right side of the lower lip, with complete healing of the former lesion by occlusive dressing (B). The follow-up interview showed, again, manipulation with the hand as well as family conflicts. Examination by a psychiatrist compared with clinical and evolutionary data showed cutaneous pathomimia.

## Image en médecine

La pathomimie cutanée est une maladie auto-provoquée dans un état de conscience claire par le patient lui-même, au niveau de son revêtement cutanéomuqueux ou de ses phanères.Nous rapportons le cas d’une jeune femme, âgée de 22 ans, sans antécédent notable, consultait pour une ulcération de la lèvre inférieure, d’installation brutale, évoluant depuis 3 mois dans un contexte de conservation de l’état général. La patiente avait consultait auparavant chez plusieurs confrères dermatologues, et bénéficiait de deux biopsies cutanées, dont les résultats étaient non spécifiques. L’examen clinique objectivait une ulcération, douloureuse, bien limitée, à surface propre, et à base non indurée, mesurant 1x3 cm de diamètre, et intéressant la moitié gauche de la lèvre inférieure (A). Devant l’aspect clinique de la lésion, son caractère chronique, et la non réponse aux soins locaux, plusieurs diagnostiques ont été évoqués: un carcinome épidermoïde, une leishmaniose cutanée, un pyodermagangrénosum… Une nouvelle biopsie profonde, objectivait une perte de substance mettant à nu le chorion, sans caractères histologiques de malignité. Les sérologies de la syphilis et du VIH étaient négatives. 15 jours après, l’évolution était marquée par l’apparition d’une nouvelle lésion identique sur le côté droit de la lèvre inférieure, avec cicatrisation complète de l’ancienne lésion sous pansement occlusif (B). L’interrogatoire répété retrouvait la notion de manipulation par la main, ainsi que des conflits familiaux. L’examen par un psychiatre, confronté aux données cliniques et évolutives ont conclu à des lésions de pathomimie.

**Figure 1:**
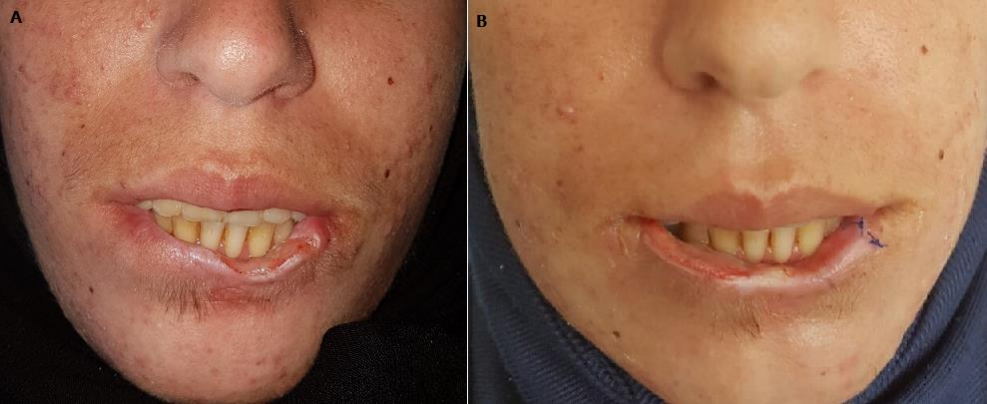
(A) ulcération douloureuse, bien limitée, à surface propre, mesurant 1 × 3 cm de diamètre, et siégeant au niveaula moitié gauche de la lèvre inférieure; (B) cicatrisation complète de l’ancienne lésion dans un délai de 15 jours sous pansement occlusif, avec apparition d’une nouvelle ulcération identique sur le côté droit

